# Exploring the validity of a bidimensional measurement model of the Screen for Disordered Eating: Compulsive/Emotional Eating and Pursuit of Thinness

**DOI:** 10.1192/j.eurpsy.2026.11367

**Published:** 2026-07-31

**Authors:** A. T. Pereira, A. I. Araújo, M. J. Brito, C. C. Marques, A. Macedo

**Affiliations:** 1 Institute of Psychological Medicine, Faculty of Medicine University of Coimbra, Coimbra; 2 Digital Human-Environment Interaction Labs, Lusófona University, Lisboa, Portugal

## Abstract

**Introduction:**

SDE was originally developed as an unidimensional measure to screen for Eating Disorders (ED) without excluding Binge Eating Disorder (BED).

It is composed of five items (yes or no), extracted from other self-reported questionnaires for the assessment of eating psychopathology. The Portuguese version of the SDE has shown good validity and internal consistency in both women (Pereira et al. 2023) and men (Pereira et al. 2024), and has been used to investigate eating psychopathology in adults. To continue our research on the role of emotion (dis)regulation in eating psychopathology we became interested in having a valid and short measure to separately assess emotional / compulsive eating and other ED symptoms.

**Objectives:**

To analyze the validity and internal consistency of a two-dimensional measurement model of SDE, grouping items 1 (emotional eating) and 2 (compulsive eating) in the same factor and items 3 (vomiting), 4 (desire to be thinner) and 5 (body dysmorphia) in another factor.

**Methods:**

830 adults from the general population (70.12% women; mean age 31.36±14.26 years; range: 18-65), completed an online survey including the of the SDE and Eating Disorder Examination Questionnaire (EDEQ-7) Portuguese versions.

**Results:**

The Confirmatory Factor Analysis of the bidimensional second order model resulted in “very good” fit indices (χ2/df=1.9515; CFI=0.9938; TLI=0.9845; GFI=0.9964; RMSEA=0.0339; p<0.001). Cronbach’s *α* coefficients of factors 1 and 2, named Compulsive and Emotional Eating (CEE) and Pursuit of Thinness (PT), were respectively .595 and .608. Pearson correlation coefficient between the two factor scores was moderate (r=0.421) and between these and the total score were high (r>0.70) (p<.001). CEE and PT dimensional scores moderately correlated with the EDEQ-7 total score (r=0.371 and 0.473, respectively; p<.001).

**Conclusions:**

SDE may be used to assess two related but relatively independent disordered eating behaviour dimensions with good validity (construct and concurrent) and internal consistency.

**Disclosure of Interest:**

None Declared

